# Supplementary material for: Antiviral type III CRISPR signalling via conjugation of ATP and SAM
Source: Nature. 2023 Oct 18;622(7984):826–33. doi: 10.1038/s41586-023-06620-5 (PMC10600005; doi:10.1038/s41586-023-06620-5)
Supplement: Supplementary file 1 — This file contains Supplementary Figures 1-10 and Supplementary Table 1 [file 41586_2023_6620_MOESM1_ESM.pdf]

nature portfolio

https://doi.org/10.1038/s41586-023-06620-5

Supplementary information

Antiviral type III CRISPR signalling via conjugation of ATP and SAM

In the format provided by the authors and unedited

Supplementary Figures

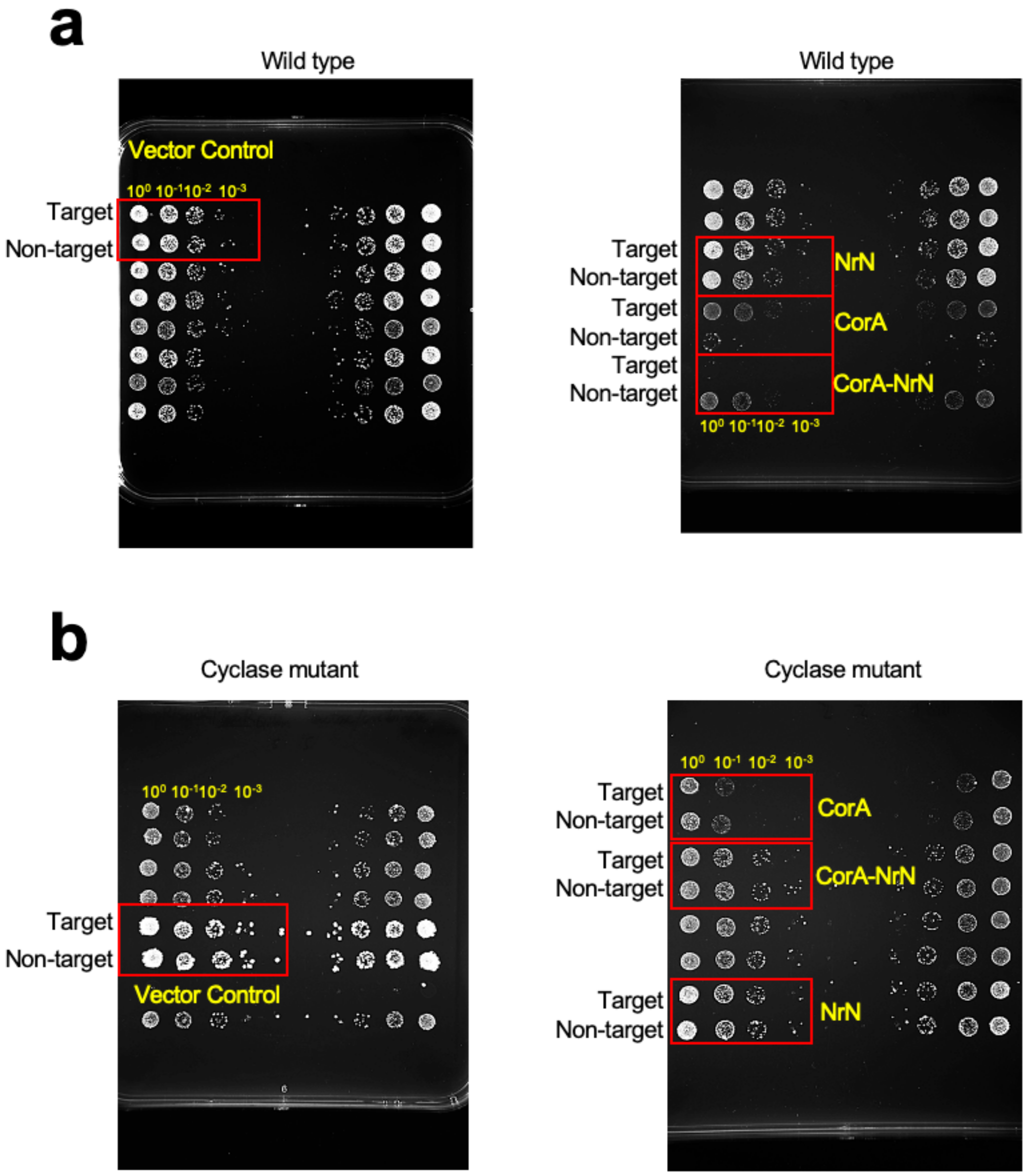

Supplemental Fig. 1.

Plasmid challenge assay of wild type (a) and cyclase mutant (b). Plate sources of Fig. 1c.

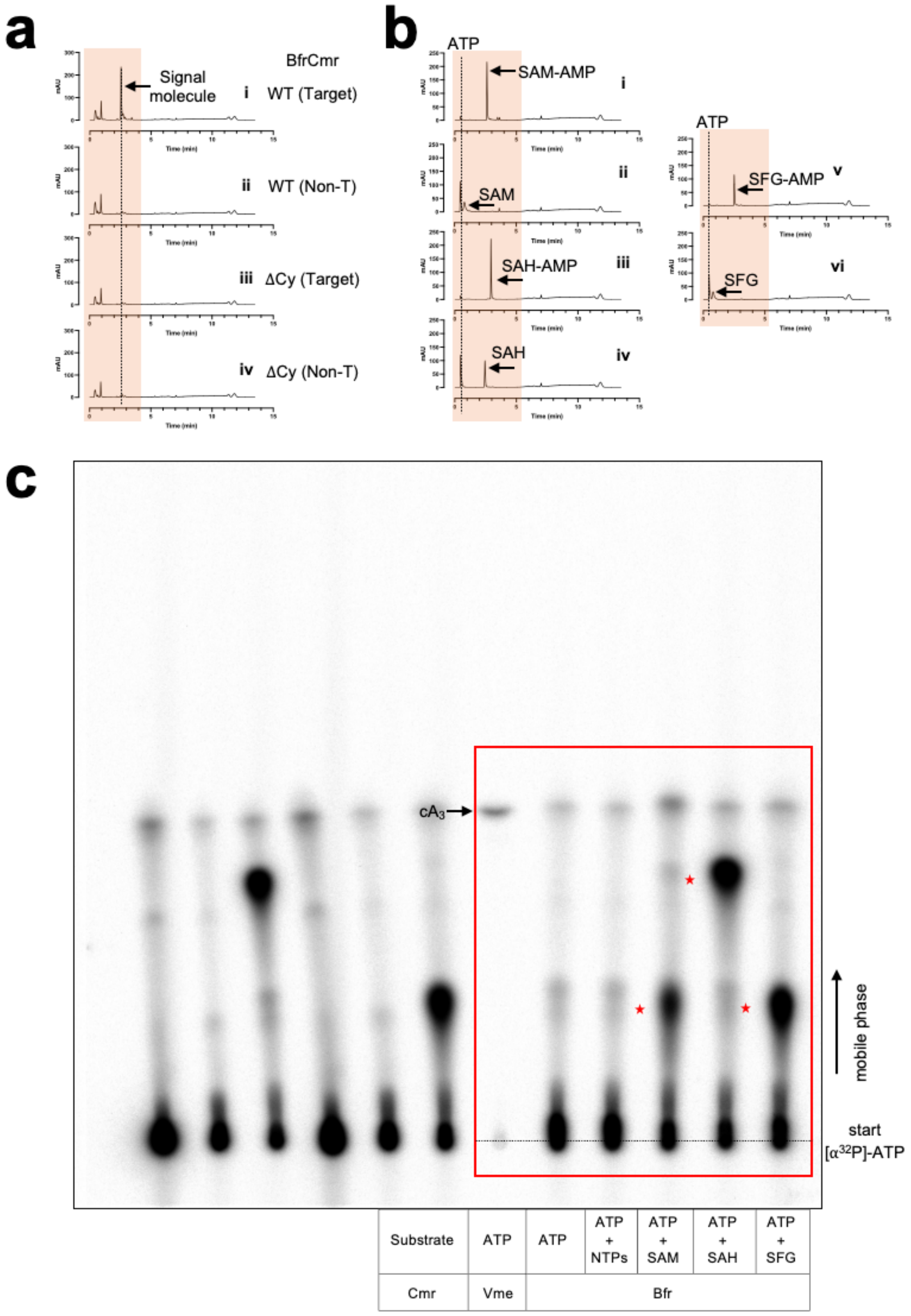

Supplemental Fig. 2.

a, and **b**, The original HPLC figures of Fig. 2a and 2e respectively. **c**, TLC image source of Fig. 2f.

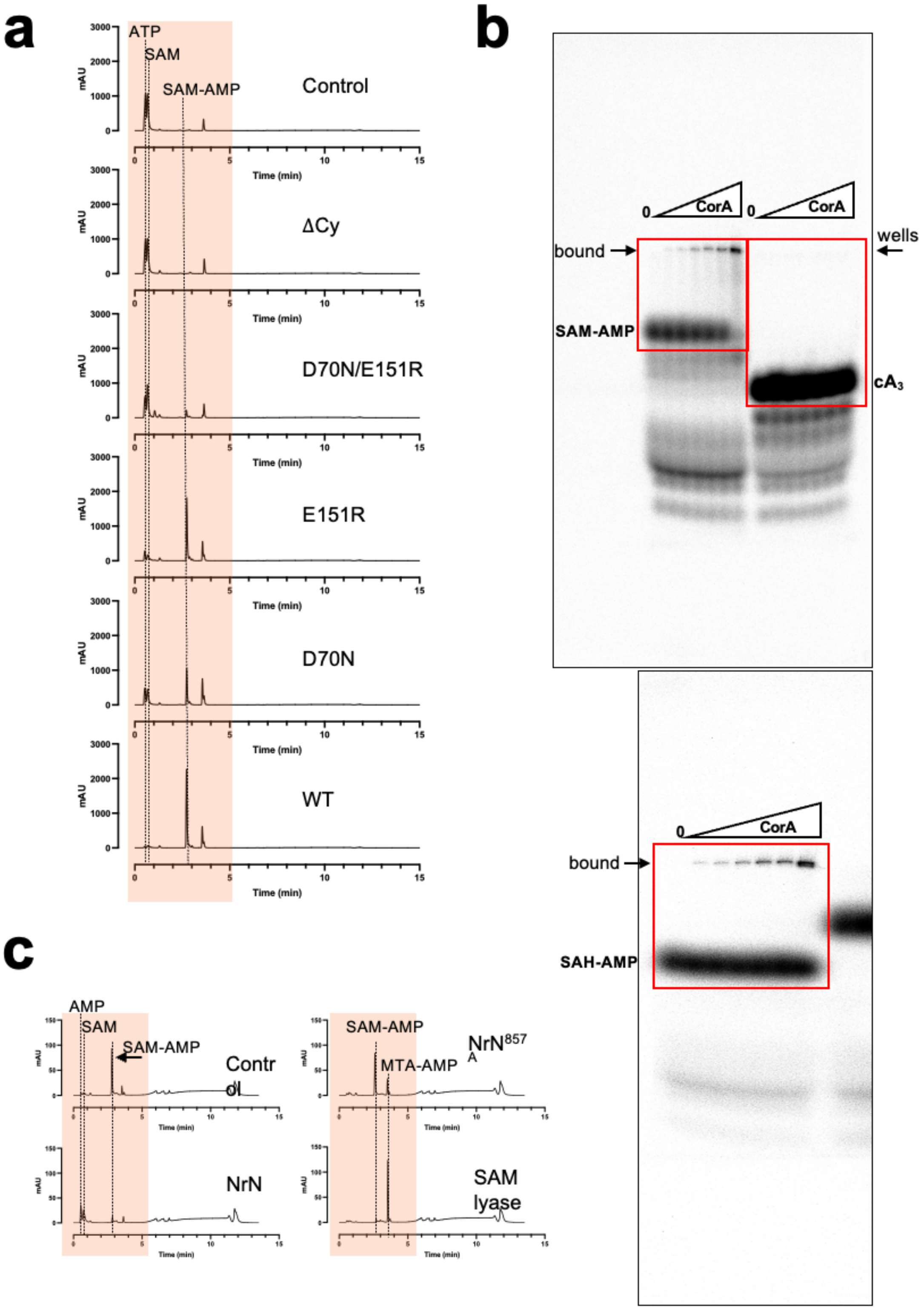

Supplemental Fig. 3.

a, The original HPLC data of Fig. 3c. **b,** Gel sources of Fig. 3a. **c,** HPLC data of Fig. 3b.

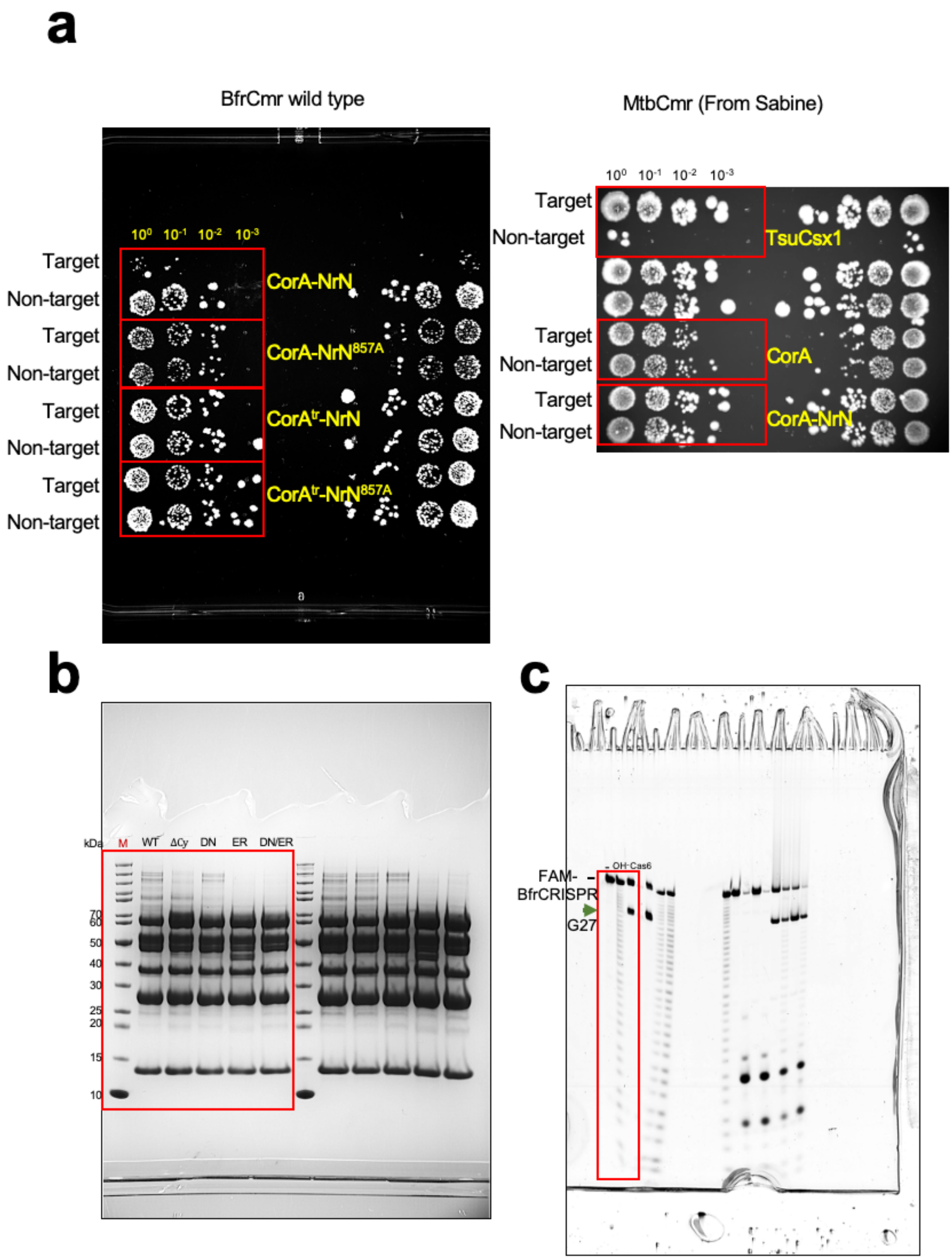

Supplemental Fig. 4.

a, The plate images of Extended Data Fig. 1b and 1c. **b,** The SDS-PAGE gel of Extended Data Fig. 2b. **c,** The original polyacrylamide gel image of Extended Data Fig. 2c.

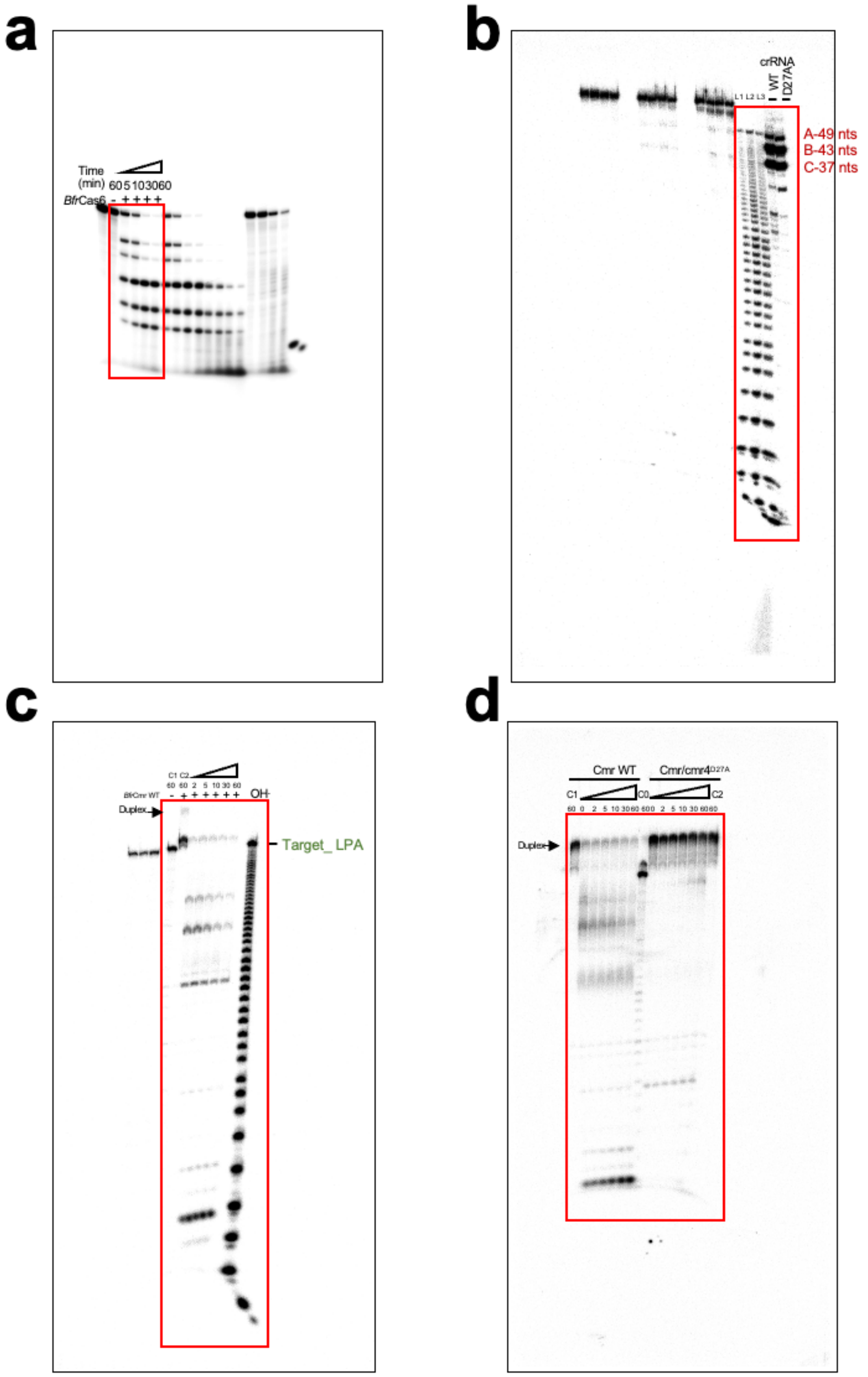

Supplemental Fig. 5.

a, b, c, and d, Gel sources of Extended Data Fig. 2c, 3b, 3c and 3d, respectively.

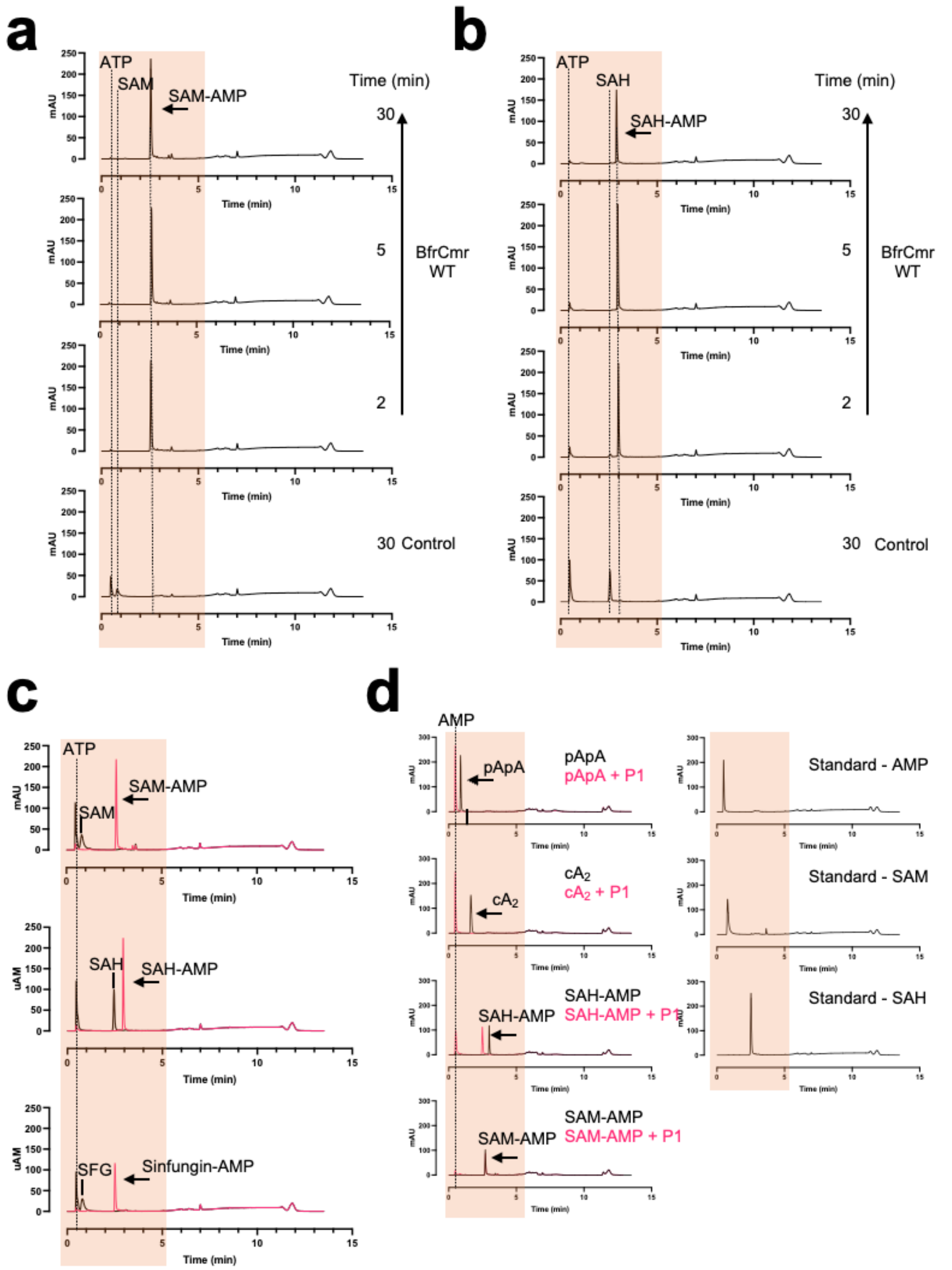

Supplemental Fig. 6.

a, b, c, and d, HPLC image sources of Extended Data Fig. 4a, 4b, 4c and 5, respectively.

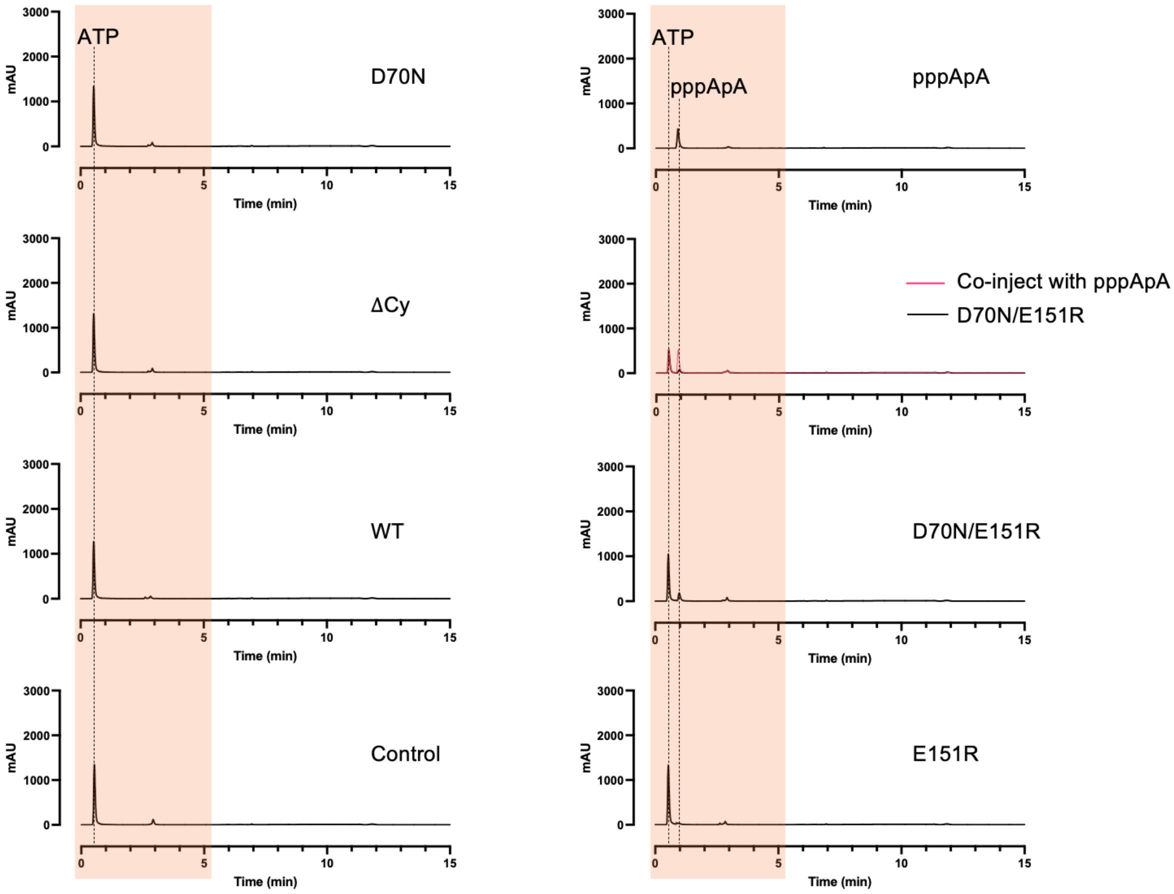

Supplemental Fig. 7.

HPLC image sources of Extended Data Fig. 7.

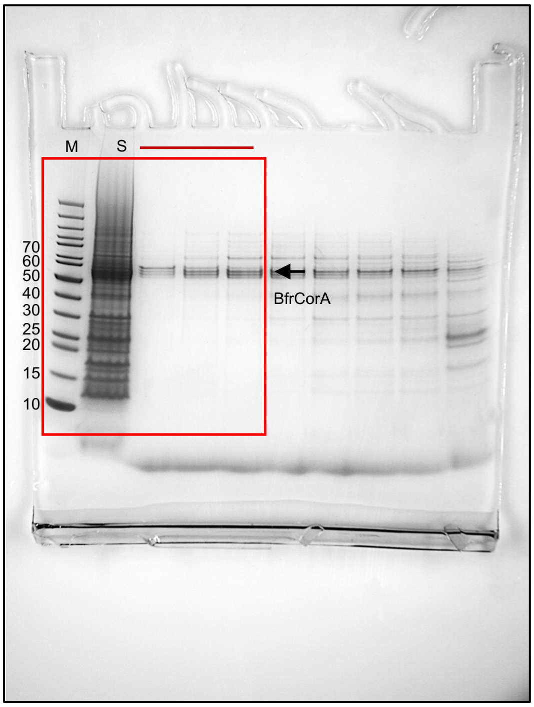

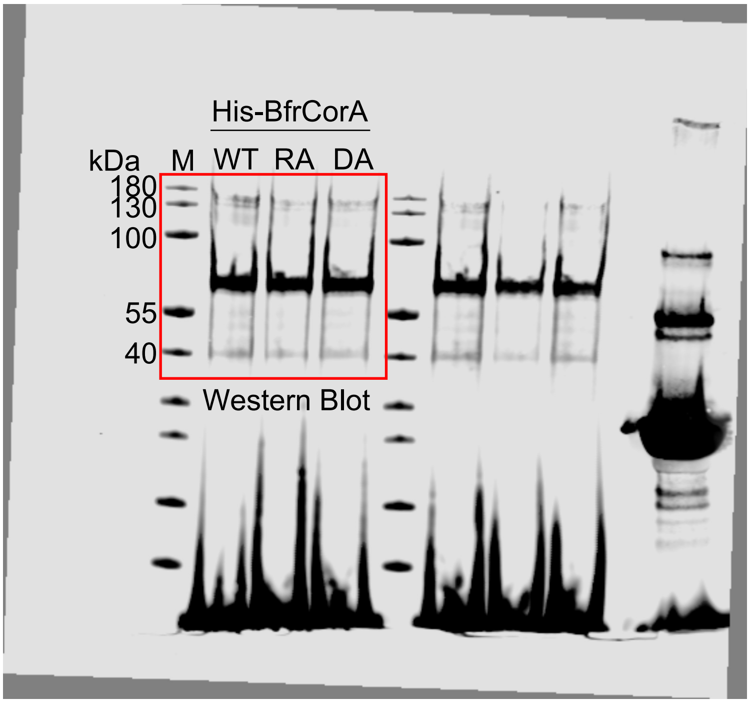

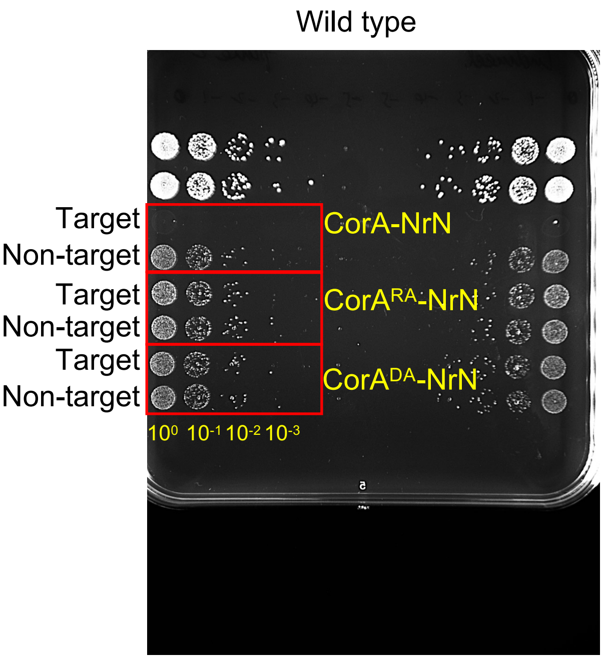

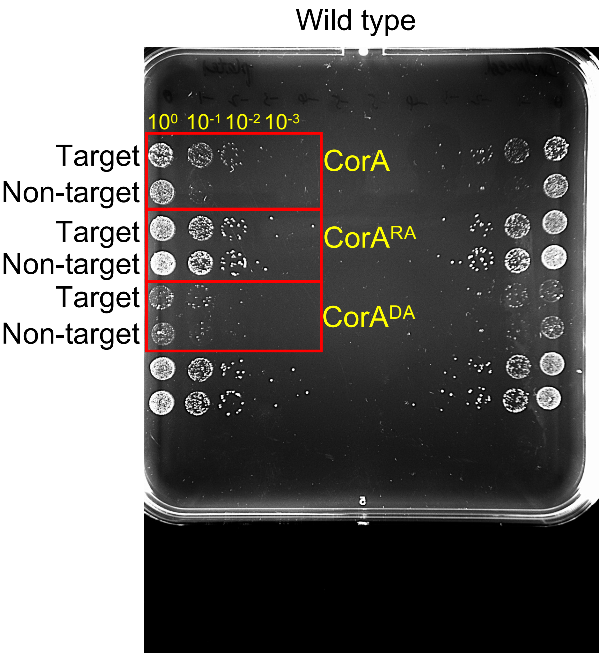

Supplemental Fig. 8
SDS-PAGE gel source of Extended Data Fig. 8a (top left). The original Western Blot image of Extended Data Fig. 8e (top right). The original plate images of Extended Data Fig. 8f (bottom).

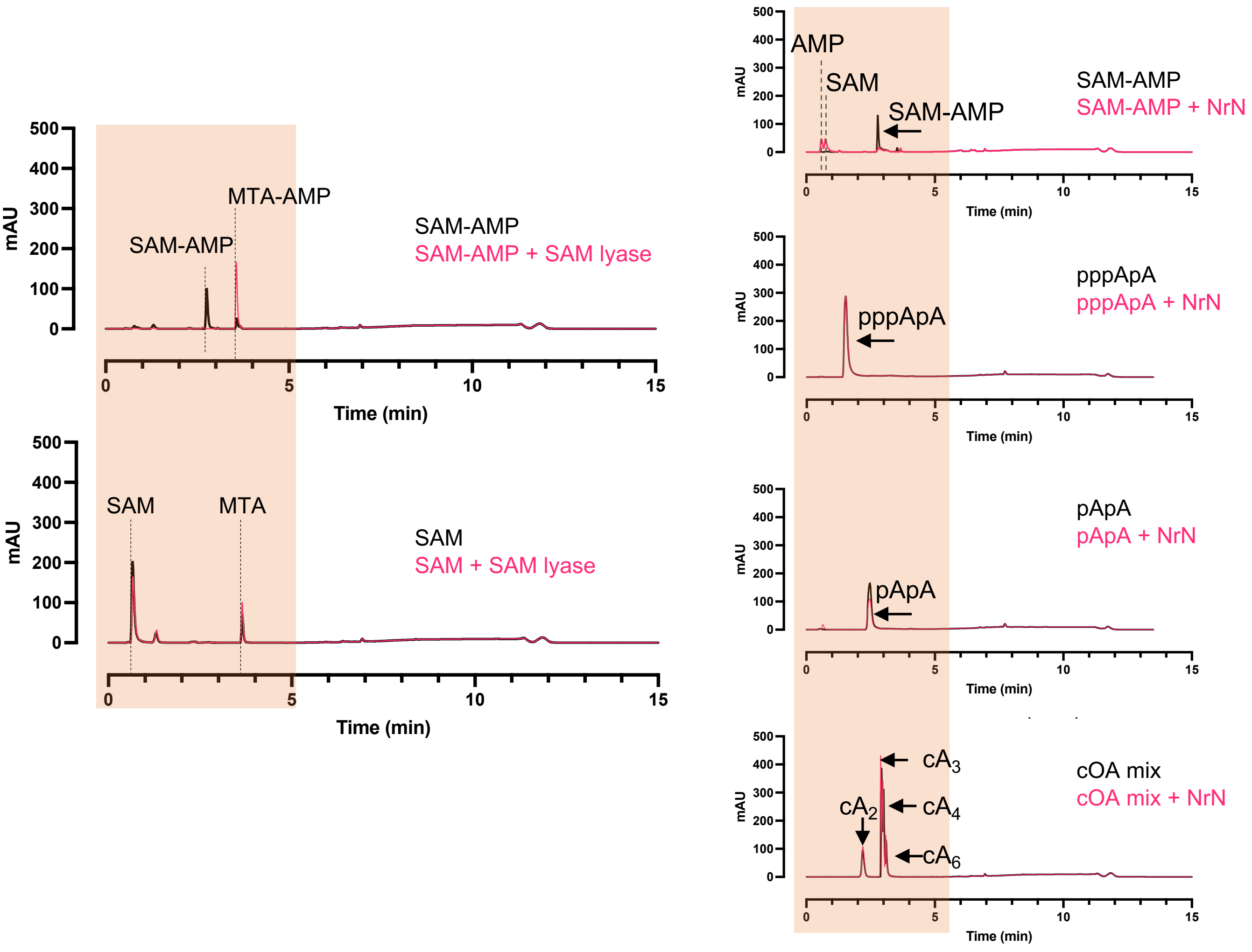

Supplemental Fig. 9.
HPLC image sources of Extended Data Fig. 9a and b.

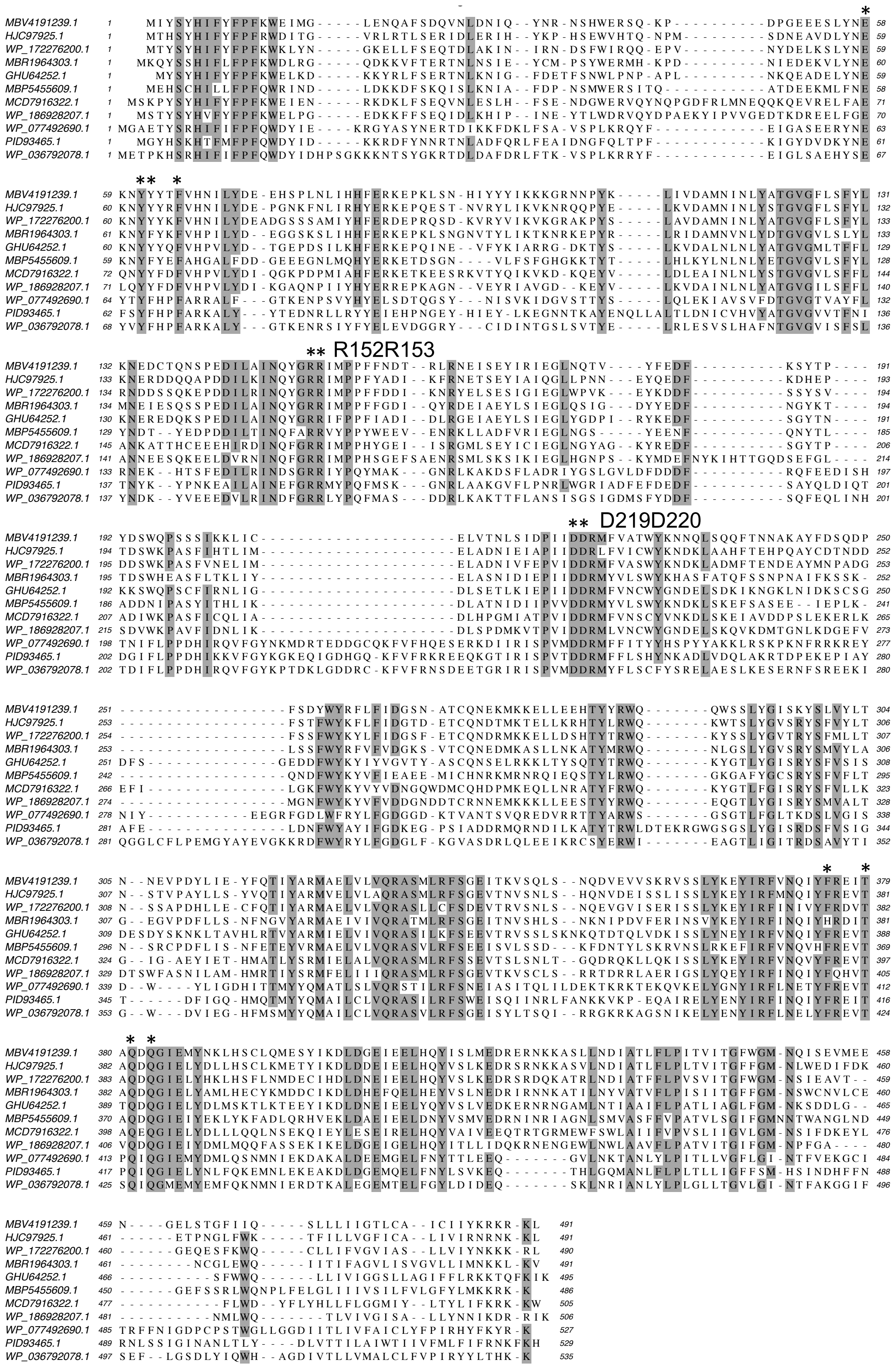

Supplemental Fig. 10.

Multiple sequence alignment of CorA proteins from Clade CorA1. Conserved residues present in the interdomain interface are indicated by asterisks, and the positions of the RR and DD motifs probed by site directed mutagenesis are shown.

Supplementary Tables

Supplemental Table 1.

DNA and RNA sequences

Name	Sequence 5' to 3'	Notes
Duet Up	GGATCTCGACGCTCTCCCT	Primer
Duet Down	GATTATGCGGCCGTGTACAA	Primer
Spacer_RNA-F	AGACGAATTCGAGCTCGGTACCCGGGGATCCTCTAG	Primer
Spacer_RNA-R	ACATCTAGAGGATCCCCGGGTACCGAGCTCGAATTC	Primer
Spacer_TetR-F	AGAC TGACGGTGCCGAGGATGACGATGAGCGCATTGTTAGA	Primer
Spacer_TetR-R	ACAT TCTAACAATGCGCTCATCGTCATCCTCGGCACCGTCA	Primer
BfrCmrSG1-f	CCAGACGTACCTGCCGGCATTCTTC	Primer
BfrCmrSG1-r	CGATTTCATTGATGCTTTCGATATTGAAGG	Primer
BfrCmrSG2-f	GCGCTGTTGTCCTTCAATATCGAAAGC	Primer
BfrCmrSG2-r	CGATTGAATCCGACCACATAAAGTTAC	Primer
BfrCmrSG3-f	CTGTCAGCTTTGTGTAACTTTATGTGG	Primer
BfrCmrSG3-r	CCCGAAATGGTTATTGAACGCGGCAAC	Primer
BfrCmrSG4-f	CGAATCGCCTCTGGTTGCCGCGTTC	Primer
BfrCmrSG4-r	CTATTTCAGCAATCTGTCTATTTCGTTCG	Primer
BfrCmrSG5-f	GCAACTATGGACGAACGAAATAGAC	Primer
BfrCmrSG5-r	GAAGTTATGACAGATGAAGAATGCCG	Primer
BfrCmr3-F	GCGCCCATGGCAATGAACCGTCATTACTTAATCACTC	Primer
BfrCmr3-R	GCGCGGATCCTTAGATATAAATGTTGTAGCCGG	Primer
Bfr-rep-5p-T	CATGGAATAGTAATCTGATTATCAATAT	Primer
Bfr-rep-5p-C	ATTATACTGGAATACATCTACATATATTGATAATCAGATTACTATTC	Primer
Bfr-rep-3p-T	ATGTAGATGTATTCCAGTATAATAAGGATTAAGACTTAAATAGAG	Primer
Bfr-rep-3p-C	TCGACTCTATTTAAGTCTTAATCCTT	Primer
Bfr-sp-phageIPA-T	ATGTAGATGTATTCCAGTATAATAAGGATTAAGACATTCGTGAGTGATTTATTTCCATG AAGTGGCGTCCCT	Primer
Bfr-sp-phageIPA-C	ATTATACTGGAATACATCTACATAGGGACGCCACTTCATGGAAATAAATCACTCACGAA TGTCTTAATCCTT	Primer
Cmr4_D27A-fw	CGGAGTTATTGcTAACTTGATCCAACGTGAC	Mutagenesis
Cmr4_D27A-rv	GGATCAAGTTAgCAATAACTCCGTAGTTCACC	Mutagenesis
BfrCas10_cyclase-Fw	CATTGGAGGGGCCGCTTTGCTTTGTTTTGCGC	Mutagenesis

BfrCas10_cyclase-RP	GCAAAGCGGCCCCTCCAATGAAGATCGGCTTTC	Mutagenesis
BfrCas10_D70N-Fw	GCAGGGTTGTTTCCCAACCGTTATATCTTCAAG	Mutagenesis
BfrCas10_D70N-RP	CTTGAAGATATAACGGTTGGGAAACAACCCTGC	Mutagenesis
BfrCas10_E151R-Fw	GTGAAAAGTACCTGAACATTATTAGAAATCAGGAGAC	Mutagenesis
BfrCas10_E151R-RP	GTCTCCTGATTTCTAATAATGTTCAGGTACTTTTCAC	Mutagenesis
NrN857A-f	TCGCCGCCGCCAATGAGTATGCCACGTATCCAAGTG	Mutagenesis
NrN857A-r	CATACTCATTGGCGGCGGCGATACGAATGTAATTGGTTGGAGG	Mutagenesis
corAtr-f	GGCGTCCTAGTTGAATGATATTGCAACTCTTTTCC	Mutagenesis
corAtr-r	GCAATATCATTCAACTAGGACGCCTTTTTGTTGCG	Mutagenesis
BfrCRISPR repeat	AUGUAGAUGUAUUCCAGUAUAAUAAGGAUUAAGAC	5’ 6-FAM™
Target RNA_Lpa	CACAAGGGACGCCACUUCAUGGAAAUAAAUCACUCACGAAUAGACACGA	RNA
Non-target RNA_pUC	AACGACUCUAGAGGAUCCCCGGGUACCGAGCUCGAAUUCCAAAGGCA	RNA
An internally radio-labelled transcript RNA	GGGGAAUUGUGAGCGGAUAACAAUUCCCCUGUAGAAAUAAUUUUGUUUAACUUUAAUAA<br>GGAGAUAUACCAUGGAAUAGUAAUCUGAUUAUCAAUAUAUGUAGAUGUAUUCCAGUAUA<br>AUAAGG/AUUAAGACAUUCGUGAGUGAUUUAUUUCCAUGAAGUGGCGUCCCUAUGUAGA<br>UGUAUUCCAGUAUAAUAAGG/AUUAAGACUUAAAUAGAGUCGACAAGCUUGCGGCCGCA<br>UAAUGCUUAAGUCGAACAGAAAGUAAUCGUAUUGUACACGGCCGCAUAAUC	T7 transcript
C. botulinum SAM lyase	GCGCCCATGGCACATATGGGGAAGACCTTACGCTTCGAGATTGTGTCGGGTGTGAATAA<br>GGGATATTTTCATACGAACTCACAGTCGGAATCACTGGACCTGGTAGGGGGTATCTGGC<br>AGAAGATCGCTAAAGAAGAATTTGAGAAATCCAATATCTACGTCAGCGCAGTTATTAAA<br>CCCAGCAAGACTGTATATAACCAGGAGTGGGGCTGTCCCGAGAATGGAGAAGAAACAGT<br>GGTGTTAACTGGAGTTGCCAATGAAGAGTTCGTTGACGATATTGAGAAATGGAAGGATA<br>CGGTAATCAAATTGGCCAAGGAGCTGAAGAACCAAATGAAACAGTCAACGTTAACGTGC<br>GAGTTTATCGAGACAGAATTGCACTACTTCAAGTGACTCGAGGGATCCCGCG	Synthetic genes
BfrNrN	GCGCCCATGGCACATATGCAAAAACAGGCGAAAGAAATCAAGAAGCATTTGTTCCTTTT<br>GGGTGGTCACGATCTTGAGATGCAGACCATTGTGCAAATCTTAACAGATCGCAACGTCA<br>TTTTCAAAGATCGTTATCTTCAATGGGACAATGCATTGTTATCGCAATACGAGGAAGAA<br>ATCCAACAATACGGGAATAAGGAACCATTCATTATTTATGGCGTCGAGCTGAAAGAAGA<br>CATTACACCTCCAACCAATTACATTCGTATCGACCACCACAATGAGTATGCCACGTATC<br>CAAGTGCCCTTGAACAGGTCGCGTCAATCTTAGACCACCCTCTGAACCGTTATCAAACA<br>CTGGTTGCTGCAAATGACAAGGCCTACATTCCGGGTATGCTTGAAATTGGAGCGAGCCA<br>TGAAGAGATTAACTTAATTCGCCAGGAGGATCGCAAAGCCCAAGGCGTTATCGAGGATG<br>ATGAGAAATTGGCGCAAGAGGCTATCACAAATGGGACTGAAAAGATTGGTAGCTTGTAT<br>GTCGTCTTTACTACCGCTAACAAATTTTCTCCGATCTGTGACCGTTTATATCCGTACGA<br>GAAATTGTTGATTTACACTCCAAATGAGTTAATCTATTATGGAAAGGGAATCAATAGTA<br>TTCAAAAGATCCTGAAGCGCTATACTCCAATCAGCAACATTTTTTGGGGCGGCGGGATC<br>AATGGCTTTATCGGGACAGTACGCAATCGCCTGACTACGAATGAGATCTTAAATATCGT<br>TGAGCAGATTAAGCTGCTGGAGCTG TGACTCGAGGGATCCCGCG	Synthetic genes
BfrCorA	GCGCCCATGGCACATATGATTTATTCTTATCACATTTTCTACTTCCCGTTTAAGTGGGA<br>GATTATGGGGTTAGAGAACCAGGCATTTTCAGACCAGGTGAACTTAGATAACATTCAGT<br>ATAACCGCAACTCACATTGGGAGCGTTCCCAGAAACCTGATCCAGGCGAAGAAGAAAGC<br>CTGTACAATGAGAAAAATTATTACTATACGTTTGTACATAACATCTTATACGACGAGGA<br>ACACTCGCCTTTGAACTTAATTCATCACTTTGAACGCAAAGAACCGAAGTTGAGTAATC<br>ATATCTACTACTATATCAAAAAAAAGGGTCGCAACAACCCTTATAAACTTATTGTGGAC<br>GCTATGAACATTAATCTGTACGCTACAGGCGTAGGGTTTTTGTCGTTTTACTTGAAAAA	Synthetic genes

	TGAGGATTGTACACAAAACTCTCCCGAGGATATTTTGGCAATCAATCAATACGGACGTC<br>GCATTATGCCTCCATTCTTTAATGACACCCGTTTACGCAATGAAATTTCTGAATATATC<br>CGTATTGAGGGGTTGAATCAGACGGTTTACTTTGAGGATTTCAAATCCTATACACCCTA<br>TGATTCATGGCAACCGAGTTCCTCTATCAAGAAACTTATCTGTGAATTGGTTACTAATT<br>TGTCAATCGATCCCATCATTGATGACCGTATGTTCGTAGCGACATGGTATAAAAATAAT<br>CAGCTTTCACAACAATTCACTAATAACGCAAAAGCATATTTTGATTCCCAAGATCCGTT<br>CTCCGATTATTGGTATCGTTTTTTGTTCATCGATGGCTCGAATGCCACATGTCAAAATG<br>AAAAAATGAAAAAAGAATTATTGGAGGAGCACACATATTATCGCTGGCAACAATGGTCA<br>TCCTTATACGGGATCTCAAAATATAGTCTTGTTTATTTGACGAATAATGAGGTGCCTGA<br>TTATCTGATCGAGTATTTCCAGACAATTTATGCACGCATGGCCGAGCTGGTCTTAGTCC<br>AACGCGCTAGCATGTTACGTTTTTCTGGTGAGATTACTAAAGTGTCACAATTGTCCAAT<br>CAAGATGTTGAAGCCGTGTCGAAGCGCGTCTCTAGCCTGTACAAGGAATATATTCGTTT<br>TGTTAATCAGATCTACTTCCGCGAGATTACCGCGCAGGACCAGGGGATTGAGATGTACA<br>ATAAACTTCACTCGTGTTTGCAGATGGAGTCTTATATCAAGGACTTGGATGGCGAGATC<br>GAGGAATTACACCAGTATATTTCATTGATGGAAGACCGCGAACGCAACAAAAAGGCGTC<br>CCTGTTGAATGATATTGCAACTCTTTTCCTTCCAATTACCGTGATTACTGGGTTTTGGG<br>GGATGAACCAGATCTCGGAAGTGATGGAAGAAAACGGCGAGCTTAGTACAGGTTTTATT<br>ATTCAAAGTTTACTTCTTATTATTGGCACGCTTTGCGCGATTTGCATCATCTATAAACG<br>CAAACGTAAATTG TGACTCGAGGGATCCCGCG	
BfrCas6	GCGCCCATGGCACATATGAAAAATACACATGTCTTACTGATTAAATTTAAGAACAAGAT<br>TAGTGACGACGAAGTTCAGTTCTTTCGTAGCTCAATCATTCAGAAGTTAGGGGACCAAC<br>CAGATATTTTATACCATAACCATGTGGAAAAGAACAAATATCGCTATTCCTACCCCTTG<br>ATCCAGTATAAAAATATCGAACAGCAAGCCACAATTGTGTGCATTGACCAGGGTACAAA<br>AGCCATTGAGAAGTTTTTCAGCCAGTGTGATTTCAACTTTCAGCTTGGTAACCGCAAAG<br>TTAATATGAAGTTCGCATCAGTGACACCCTATAAGTTGCTGATCGAACGTCAATCACGC<br>ATGATTAATTACCATATTCATAACTGGTTGCCCTTGAACTCTGACAACTATAAGAAGTA<br>TCAAAATATTAGTATCTTGTCAGAACGCATCAACTTCCTTGAGAAAATCTTAGTTGGCA<br>ACATTCTGTCATTTACCAAGGGAGTAAATTATTTCATTGACTTTCCTTTGCAGTGCAAG<br>TTGCTGCAGCTTTCTTTTGCCAAGTTAATTTCTAATAAAAACATCAAGTTGATGAGCTT<br>TGATGCGGACTTCCAATGCAACTTGAACTTACCCGACTATATTGGTATTGGCAAACACA<br>CATCTATCGGCTATGGGACGATCACTCGCAACTGACTCGAGGGATCCCGCG	Synthetic genes
CRISPR pre-array	CGCGCCATGGTAAAAATACAATTTTTACCCTAACTGACTGTTGTAACTTACTTTTATAG<br>ATTTATTCTATAATGTAGATGTATTCCAGTATAATAAGGATTAAGACGTGTCTTCGTAC<br>CTTGAAGACCAATGTAGATGTATTCCAGTATAATAAGGATTAAGACAATCTTTATATAT<br>CTTATGGTTGCAGATCTAAAAAGTTGGGATTATATAAATGACAGTCGACGCGC	Synthetic genes
BfrCmra	CCAGACGTACCTGCCGGCATTCTTCATCTGTCATAACTTCGGGACCCGTAATAATAAGG<br>GATTCGGTAGCTTCACGGTGGAGTACATCAATAACCAAAAAAATATCTGTAATGTCGAG<br>GACACATTGAAAGAAAATTTTGCGTTCGTATATAAGAAAAAGATCGCTCTTTCGCGTCA<br>ATCCACACTGGACTTTATTTATATTTATAATCAGATCTTTAGTACAATCAAAAAGGACT<br>ATCAAATTCTTAAGAGTGGCTATAATTTTCGTAATGAGTATATCAAATCCTTGCTTTTT<br>TGCTACTTTGTGTCCAAGTATCCAAATTATCGCTGGGAAAAACGCAAGATGAAACAGCT<br>TATTAAGGCCCGTGGCTATGAATTGAAAGGAGATCATTCGCCAATCAGTGGGATTCGTG<br>AAAACGACAATTCTTGGAACGACCCTAATCCCAACGGGTATAATTATGCGTATATTCGT<br>GCTATTCTTGGCCTTGCTGAGCAGTACGAGTTCCAGTTGGAAACACCCTACCAGAAGGC<br>AATTGTTAAAATCAAGTCGGCCAATAACTGCATCTCACGTTATAAATCCCCTTTACTTT<br>TCAAAATCATTAATAACTCCATCTACTTGGTGGGGAACGAGATCAATACGGAAATTTTG<br>AATAAGCCGTTTCAGTATTCATACATTGAACAAACGAAAAACAAGAACATGCGCACTGG<br>AAAGTCAGAGATTACGGAACGTACAATGCATATCAATGAAATCGAGATGAACTACAAGA<br>ACCGTATTAACTATCACTACACTCCAACAAGCTTTTCATTGATCGACTTTATGCAATAC<br>GCGATGTCATACAAGAAGAACGGTAAAAACATCTTAAATTATATTCCCTTAAAACAGTA<br>AGACTAAGAAGGAGATATACATATGAAATACATCGCGATCACTCTTGGTCCGATTACTC<br>GCACCATCGAGATGGCAGAATCCACGAAGGAGTTGTGGGCGGCGTCTTACTTTTTTTCG<br>TATCTTGCCAAGAAAATTGTAGAACCCTTTGTCAAAAAGAATCGCACGTTTCAATTACC<br>TCTTATTAACGAGGAGATGCAGAAGCCCCACTGCGGTGCAGGGTTGTTTCCCGACCGTT<br>ATATCTTCAAGTCGGAACCTGGAGACCTGGAGTTACTTAAGCAACATTCCGACCAAGTA<br>CTTATCGAGATCGCGGGCCATATCGCGAGCCCCAGTTTACCTGGGACAGCGAAAGATGT<br>GTCGCAAATTTACCATTACCTGAAGAGTTATATCAAGATCTATTTCATCGAGCGCACAC<br>TGGAATCCGATGACCCTCATGTAGTCATCCCGGCCTGTGAAAAGTACCTGAACATTATT<br>GAAAATCAGGAGACTTTTCCGGAGCAGGAGGAAACCATGATTTCCCACCAGAAAAGTGA<br>TTTCCTTAAATTCTTAATTACAAACGTTAATGGTAAAATCTACCGCAAAGACAAGAATA<br>GTATTCCACGCTTTACTGGCTCATTCTTGACTCGCGACGCTTTCGGAGACATGAATGGA<br>GAGCGCCTTTTTGAGAGCATCTTAGAAATCTCTGCGAGTGAGCTTAACATTAACATTCA<br>GCAGAAGGCGTTGGAGGTTATCACTGCAAACGAGAAGAACAAAGGCGAAAAGTATAGTG<br>ACCAAATCTGGGACGCAGAAGAAATTATCCTTAACGATAACAAAGCACAATTACGCCCC<br>TACCACAAATACATCGCTATTATTAAAAGTGACGGAGATTCTATGGGAGAAACGATTAA<br>GAGCATGGGTGCATACAACATCCCAATTACTCAGCTTTCAAAAGCGCTGTTGTCCTTCA<br>ATATCGAAAGCATCAATGAAATCG	Synthetic genes
BfrCmrb	GCGCTGTTGTCCTTCAATATCGAAAGCATCAATGAAATCGTTGCCTATGGCGGAAAGCC<br>GATCTTCATTGGAGGGGACGATTTGCTTTGTTTTGCGCCGGTATGTTGCAACGGTAATA<br>ACGTTTTCAATTTGGTCGAGAAACTGAGCACTTGTTTCGACCAGTGTATTAATCAACAT<br>CTTCAACAATACATTAATGCTTGCAGCGAGGCGCAGCGTCCCTTACCAAGCTTGTCTTT<br>CGGTATCAGCATCACGTATCATAAATACCCTATGTTTGAAGCCCTTCACACTACCGACT<br>ATCTTTTAGAAATGGTGGCCAAGGACAACTTGTTCAAGTATACCTTGAGCAATAAAAAC<br>ATTCTGAATGAAAATATGAAGCGCTTTATTTTGAAAAATAAATTGGCGTTCTCTCTTCA<br>AAAGCATAGTGGACAGATCTACCATACCGCTATGTCGAAAAAGGGAAAGTCCTACGTGA<br>AGTTTAACATGCTTCTTCAAAAGTACATTCTGAAGAACAAGGACATGAGTAAGACCCAG	Synthetic genes

	GAATCTGAAAAATTTTTATCATCCGTAATCCAAATGATTCGTGCTCATGCTGAGATCCT<br>TCAAATCATTTTGCAGAATGAAGACAAACGTACCGAAATGTTAAAAAACTACTTTGATA<br>ACAACTTCAATGAGAGTTGTCACCTTGGGTACACGGGATTGTTTGAGGATATCCAAACC<br>TTGCTGTGTTTACGCTACCAAGAAAATATTCAAGATTACCAAAACCGTAATGAAATTAT<br>TCAGCAGAACACTATCCTGACGAGTGACGAGAAGGAGATTCTGATCGTGTCACCGGCCA<br>TGGATGCAATTCATACGATTTTCACAGCGTTGCAATTTATCCACTTCATTAATTATAAT<br>AAAGATGAGTAACCTTAAGAAGGAGATATAACCATGTCGCATCACCATCATCACCATCA<br>TCACGATGGCAAACCGATTCCGAACCCGCTGCTGGGCCTGGATAGCACCGGCAGCGACC<br>AGACCGAGAACAGCGGCGAAAACCTGTATTTTCAGGGCGCAAACGCCATGAACCGTCAT<br>TACTTAATCACTCTGACCCCGATGGATTGGTTTTTTTTGGCGGTGAGCGTACGCTGGA<br>TGACGGTAAGTCCGCTGACTATATCAGCCATTCCAACAAGTTTCCTCAGCAGTCTGCCC<br>TGTTAGGGATGATTCGTTATCAGTTGCTTAAACAGCATAACTTATTGTCACAATTCCCA<br>TATACAGAAAATAAGCCCACCGAGAAAGAGATTATGAAAACCCTGATCGGGGAGCAAAG<br>CTTCCGCATGACAGAGCGCAAGGCGAAAAGTCTGGGATTGGGCGTAATTAAACAGATTA<br>GTCCATTAATGCTGATCGAGTGTAAAGATGACACTTCCTCCCGTAGTATCTATTTCCCC<br>TTACCTCTGGACGACGGTTATAAGGTGTCGTTCAATGAGACGAGCAATGAGGACAAAGT<br>GTTCTATAACGGGATTGAATGCCCCATTCCTAACGTATATCCTGCATCGGAGGAGCAGG<br>ACAGCGGGAATCAGAAACGCAAGTTTTTCGATCATAAAACATACAACAACTATCTGTTT<br>TGGTGCACTCAAGGAAACAACCAGATTAAGAAGCTGTTGTCAGACGAGATCTGGATTTC<br>TAAGATGCAAATCGGCATTACAAAGCACGTTGAAGAAGGAGAGGATAACGATAAATCGT<br>TTTATAAACAAGAGTTTCTGCAGCTTAAAAAGAGCTTCATCTATGCCTTCTATATCACA<br>TTATCGGGCGAAAGCGAATTGTCATCGGACATTATTCAACTTGGCGGTCAACGTTCCGT<br>ATTTCGTATGGAGGTTGAAAGTATTGAAGAAAATTCCGACATTCAGGAGAAATATCAGA<br>CTGCAGCACAGTTTTTGACCCAGTCCGACCGTCTTCTTATCCTTTCTCCTACGTATGTA<br>GACAATTTAAAAGAACTGTCAGCTTTGTGTAACTTTATGTGGTCGGATTCAATCG	
BfrCmrc	CTGTCAGCTTTGTGTAACTTTATGTGGTCGGATTCAATCGTATTTCGCAATATCCAAAC<br>GACGAATGCCTCTAACTTCTATGGGAAACCTATCAAATCGTCCTCCAAATATCACTTTT<br>TAAAGCCGGGGTCGGTCTTATATTTCAAACAAGGCAAACGTAAAGAGGTTGAGAAGCTG<br>CTGATGGATTATACTTATCTGCGCCTTTCCGGCTACAACATTTATATCTAAGAACAGAA<br>AGTAATCGTATTGTACACGGCCGCATAATCGAAATTAATACGACTCACTATAGGGGAAT<br>TGTGAGCGGATAACAATTCCCCATCTTAGTATATTAGTTAAGTATAAGAAGGAGATATA<br>ACCATGACCACACGTATGTACGTCATTAACACGTTAAGCAATATGCACGTGGGTAGCGG<br>GGAGGTGAACTACGGAGTTATTGATAACTTGATCCAACGTGACTCTGTAACGAACTTAC<br>CAAACATTAATTCTTCGGGGTTAAAAGGCGCGATTCGTGAATACTTCAAGGAGAATGAA<br>GACCTGGTACGCGAGTTGTTCGGATCAGCTCCACGTGACGAGAAGACGTTGCCAGGAAA<br>AGTCCGCTTTTTCGAAGCGAACTTACTTTCGATGCCGGTCCGCTCCGATAAGGTCCCCT<br>TTTTGATGGCTATCAGTGATGAGGTATTGCAAGAGCTGATTACCAAAATGAAATTCTTT<br>AATTGTGAAGAGGCGACTCAGTACATTTCCCATTTGAGCACTTTACTTGATAACATTAA<br>AACACAAGCGCAAGGTACCGACTTTGCCTACGTCTTTGACCCTTTATTGCAGGGTGCTA<br>TCATTGAGGAAGTATCGATCCGTGCAACTTGCCCGTCGCACATCCCTCTTCAGCCTTCA<br>CTTAAGAAATTACTTGGTGATCGTCTTGTGATCTTGTCCCATAAGTATTTTTCAATTTT<br>GAGTGATGATAATCATTTGCCGGTTCTTTCTCGCAACAATCTGGAGAACGGGCAGTCAG<br>CCAATCTTTGGTATGAGCAAGTGCTGCCGCGTTATAGTCGCTTGTACTTCATGTTGATG<br>GATGGTAATGCTCAATCCGAGTACCTTAAAAAAATTTCGTGACACATTGTGTACCCCGTC<br>TACCATCATTCAGATTGGAGCTAATGCGTCGATTGGATATGGGTATTGCCAAATTAGCG<br>AACTGTCGCCGTTCTAACTATAAGAAGGAGATATACACATGAAGATTAGTAAGAAACAA<br>ATCGAATATGCCATCGAAGCACTGCGTGCAAATAACATTATCACAAATGACAACCAGTA<br>CCCGAAGGTTTTCAAAGGATACATTTCTTCCTTCGGCGCGGCAGTGATCCAGAGCGGAC<br>TTATTCCTGCAATCATTTTTTTCGAGAACGAAGATAACGACGCCAACGCGGACCGTCAC<br>AAGATTATTGGTGTGTTGAAGGACATTATCAACGCAATGCGTCAGCAATACACAGTTAC<br>TGATGCGACTATTTTGGTCTCTTCACAAATTCCCGCAAATTATAGTATGGCGCAGTACA<br>TTATTGAACATGGCAATACGGATCAATTACTTAAGGAAATTACAGAGGCTGCTGTGGCA<br>ATGAAGTTAGCGTTACGTATGTATAAGAGCGAGTGAGTCTAAGAAGGAGATATAACTAT<br>GCCGAAAAATTATACGTTACAAAATGCGTCAAACCTTGGATGGCTGTTCTACAAGGATT<br>ACTACCGCCAGGAACCAAACGTAGACTTCATCTCGACTCAGGGTAAGGAGTCCGATACC<br>ACGGCCGACTTTTTTCGTAAGACAAACCAGCGTATTACGGCCTACCAGCTGAATAGCGA<br>ATCGCCTCTGGTTGCCGCGTTCAATAACCATTTCGGG	Synthetic genes
BfrCmrd	CGAATCGCCTCTGGTTGCCGCGTTCAATAACCATTTCGGGACTCCCCTTCAACTGAAGA<br>CCATTTATCCTGGGTTGATCACAGGGTCGGGTCTGCCACACCAAACAGGGTCCAAAGGC<br>GAATTTAAACTTGGATTCCAATTTGACTATACGACGGGCTTACCCTACATTCCCGGAAG<br>TAGTATTAAAGGAACTCTTCGCTCTATGTTCCCGTTTTCGCTTAAGGACAAAGGTTCTA<br>CAAAGCGTATTTTACCAGAGTATCGCAAGGAACGTATGGAATACATCCGTGACTTGATT<br>ATCGAGGTAACCAACATTAACGAAATTTCTGACACGGAAATTCAAGCTCTGGAATACGC<br>TATCTTCACCAATTCCACACCGTCTGGAAAAACAATTGAGTTTAGCTTAGAGGAAAAAG<br>ATGTTTTCTATGACGCGTTCGTCGCTGATTCCAAAGATGGGGTAATGCTTTCAGATGAT<br>TATATTACTCCTCACGGGGAGAATCCCCTGAAAGATCCAAAACCCATTTTGTTCTTGAA<br>GATTCGCCCGGATGTAACAATCAACTTCTACTTTAAGTTGTGCACAACACACTTGTATA<br>AGGAAAAAGTCTGTAGCTCCAAGCAGATCGAAGAGATTAAGAAACAAAATGATTTTAGT<br>TCCAGCGATTACAAAATGATTACGGCCCATCAGAAGCGCAACTTGTTCGAGAAGATTTT<br>ACTTTGCATTGGTATTGGGGCTAAAACTAACATTGGATACGGGCAGTTGAAGAAATTAT<br>GACCTGTAAAACGACGGCCAGTGAATTCCCCGGGAAGCTTCGCCAGGGTTTTCCCAGTC<br>GAGCTCGATATCGGTACCAGCGGATAACAATTTCACATCCGGATCGCGAACGCGTCTCG<br>AGAGATCCGGCTGCTAACAAAGCCCGAAAGGAAGCTGAGTTGGCTGCTGCCACCGCTGA<br>GCAATAACTAGCATAACCCCTTGGGGCCTCTAAACGGGTCTTGAGGGGTTTTTTGGTTT<br>AAACCCATCTAATTGGACTAGTAGCCCGCCTAATGAGCGGGCTTTTTTTTAATTCCCCT<br>ATTTGTTTATTTTTCTAAATACATTCAAATATGTATCCGCTCATGAGACAATAACCCTG<br>ATAAATGCTTCAATAATATTGAAAAAGGAAGAGTATGAGTATTCAACATTTCCGTGTCG	Synthetic genes

	CCCTTATTCCCTTTTTTGCGGCATTTTGCCTTCCTGTTTTTGCTCACCCAGAAACGCTC<br>GTGAAAGTAAAAGACGCAGAGGACCAATTGGGGGCACGAGTGGGATACATAGAACTGGA<br>CTTGAATAGCGGTAAAATCCTTGAGAGTTTTCGCCCTGAAGAGCGTTTTCCAATGATGA<br>GCACTTTCAAAGTTCTGCTATGTGGAGCAGTATTATCCCGTGTAGATGCGGGGCAAGAG<br>CAACTCGGACGACGAATACACTATTCGCAGAATGACTTGGTTGAATACTCCCCAGTGAC<br>AGAAAAGCACCTTACGGACGGAATGACGGTAAGAGAATTATGTAGTGCCGCCATAACGA<br>TGAGTGATAACACTGCGGCGAACTTACTTCTGACAACCATCGGTGGACCGAAGGAATTA<br>ACCGCTTTTTTGCACAATATGGGAGACCATGTAACTCGCCTTGACCGTTGGGAACCAGA<br>ACTGAATGAAGCCATACCAAACGACGAGCGAGACACCACAATGCCTGCGGCAATGGCAA<br>CAACATTACGCAAACTATTAACTGGCGAACTACTTACTCTGGCTTCACGGCAACAATTA<br>ATAGACTGGCTTGAAGCGGATAAAGTTGCAGGACCACTACTGCGTTCGGCACTTCCTGC<br>TGGCTGGTTTATTGCTGATAAATCTGGGGCAGGAGAGCGTGGTTCACGGGGTATCATTG<br>CCGCACTTGGACCAGATGGTAAGCCTTCCCGTATCGTAGTTATCTACACGACGGGTAGT<br>CAGGCAACTATGGACGAACGAAATAGACAGATTGCTGAAATAG	
BfrCmre	GCAACTATGGACGAACGAAATAGACAGATTGCTGAAATAGGGGCTTCACTGATTAAGCA<br>TTGGTAAACCGATACAATTAAAGGCTCCTTTTGGAGCCTTTTTTTTTGGACGGACCGGT<br>AGAAAAGATCAAAGGATCTTCTTGAGATCCTTTTTTTCTGCGCGTAATCTGCTGCTTGC<br>AAACAAAAAAACCACCGCTACCAGCGGTGGTTTGTTTGCCGGATCAAGAGCTACCAACT<br>CTTTTTCCGAAGGTAACTGGCTTCAGCAGAGCGCAGATACCAAATACTGTCCTTCTAGT<br>GTAGCCGTAGTTAGGCCACCACTTCAAGAACTCTGTAGCACCGCCTACATACCTCGCTC<br>TGCTAATCCTGTTACCAGTGGCTGCTGCCAGTGGCGATAAGTCGTGTCTTACCGGGTTG<br>GACTCAAGACGATAGTTACCGGATAAGGCGCAGCGGTCGGGCTGAACGGGGGGTTCGTG<br>CACACAGCCCAGCTTGGAGCGAACGACCTACACCGAACTGAGATACCTACAGCGTGAGC<br>TATGAGAAAGCGCCACGCTTCCCGAAGGGAGAAAGGCGGACAGGTATCCGGTAAGCGGC<br>AGGGTCGGAACAGGAGAGCGCACGAGGGAGCTTCCAGGGGGAAACGCCTGGTATCTTTA<br>TAGTCCTGTCGGGTTTCGCCACCTCTGACTTGAGCGTCGATTTTTGTGATGCTCGTCAG<br>GGGGGCGGAGCCTATGGAAAAACGCCAGCAACGCGGCCTTTTTACGGTTCCTGGCCTTT<br>TGCTGGCCTTTTGCTCACATGTTCTTTCCTGCGTTATCCCCTGATTCTGTGGATAACCG<br>TATTACCGCCTTTGAGTGAGCTGATACCGCTCGCCGCAGCCGAACGACCGAGCGCAGCG<br>AGTCAGTGAGCGAGGAAGCGGAAGAGCGCCTGATGCGGTATTTTCTCCTTACGCATCTG<br>TGCGGTATTTCACACCGCAATGGTGCACTCTCAGTACAATCTGCTCTGATGCCGCATAG<br>TTAAGCCAGTATACACTCCGCTATCGCTACGTGACTGGGTCATGGCTGCGCCCCGACAC<br>CCGCCAACACCCGCTGACGCGCCCTGACGGGCTTGTCTGCTCCCGGCATCCGCTTACAG<br>ACAAGCTGTGACCGTCTCCGGGAGCTGCATGTGTCAGAGGTTTTCACCGTCATCACCGA<br>AACGCGCGAGGCAGGGGGAATTCCAGATAACTTCGTATAATGTATGCTATACGAAGTTA<br>TGGTGTCCGGGATCTCGACGCTCTCCCTTATGCGACTCCTGCATTAGGAAATTAATACG<br>ACTCACTATAGGGGAATTGTGAGCGGATAACAATTCCCCTGTAGAAATAATTTTGTTTA<br>ACTTTAATAAGGAGATATACCATGAACCAACTTACTGCCATTTTGAAGCAGCATACCCC<br>AATGATTCATTTCCAGCACAACGAGTCAGGGGCTACCCTTCGTGCGTCCGAGGTTAAAC<br>CCCTGCTTGATAAGTTTATTTTAACTAAGCTGGGGAATGGAGACATCCGCGAGGGACGT<br>TTGTATGCTAAAAAAAATAATTGGTTGATTGACAATGAGAAGAACTACGCTCTGAATTA<br>CAAATTGTCAATTTCGCTGCAGAAGAAAAGCCGTCTGGAATACCTTATCACCTCTAGCA<br>CATTTCCTTTGCCAACAGAACGTCCGTCAAATTTCTTTACCATCCAAAACTCTCCTTAC<br>TTTGCACAAGAAAAGTGCGTCGGAATTAATACGAACAGCACCATCATTTTGAAGAAGTC<br>AAACTCGGACCCGCGCAAAAAGGAGGCGGAGTTCAAAGAGAAAAACTGGTCACAGATTG<br>ATAAAAAGGGTCTGGAATGGCAAGATTTTACTATTAAAATCTTCTCTCTTAAGGGGGAT<br>TTAATCAATAAAATCCAGACGTACCTGCCGGCATTCTTCATCTGTCATAACTTC	Synthetic genes
