# Supplementary material for: Antiviral type III CRISPR signalling via conjugation of ATP and SAM
Source: Nature. 2023 Oct 18;622(7984):826–33. doi: 10.1038/s41586-023-06620-5 (PMC10600005; doi:10.1038/s41586-023-06620-5)
Supplement: Supplementary file 2 — Reporting Summary [file 41586_2023_6620_MOESM2_ESM.pdf]

nature portfolio

Corresponding author(s): Malcolm F White

Last updated by author(s): Aug 16, 2023

Reporting Summary

Nature Portfolio wishes to improve the reproducibility of the work that we publish. This form provides structure for consistency and transparency in reporting. For further information on Nature Portfolio policies, see our Editorial Policies and the Editorial Policy Checklist.

Statistics

For all statistical analyses, confirm that the following items are present in the figure legend, table legend, main text, or Methods section.

n/a	Confirmed	
☐	☒	The exact sample size (*n*) for each experimental group/condition, given as a discrete number and unit of measurement
☐	☒	A statement on whether measurements were taken from distinct samples or whether the same sample was measured repeatedly
☒	☐	The statistical test(s) used AND whether they are one- or two-sided<br>*Only common tests should be described solely by name; describe more complex techniques in the Methods section.*
☒	☐	A description of all covariates tested
☒	☐	A description of any assumptions or corrections, such as tests of normality and adjustment for multiple comparisons
☐	☒	A full description of the statistical parameters including central tendency (e.g. means) or other basic estimates (e.g. regression coefficient) AND variation (e.g. standard deviation) or associated estimates of uncertainty (e.g. confidence intervals)
☒	☐	For null hypothesis testing, the test statistic (e.g. *F*, *t*, *r*) with confidence intervals, effect sizes, degrees of freedom and *P* value noted<br>*Give P values as exact values whenever suitable.*
☒	☐	For Bayesian analysis, information on the choice of priors and Markov chain Monte Carlo settings
☒	☐	For hierarchical and complex designs, identification of the appropriate level for tests and full reporting of outcomes
☒	☐	Estimates of effect sizes (e.g. Cohen's *d*, Pearson's *r*), indicating how they were calculated

Our web collection on statistics for biologists contains articles on many of the points above.

Software and code

Policy information about availability of computer code

Data collection	Phosphorimage data were collected using GE Healthcare ImageQuant TL 7.0<br>HPLC data were collected using Chromeleon 6.8 Chromatography Data System software (ThermoFisher).
Data analysis	Graphpad Prism 9 was used for data analysis. Bioinformatic analysis was carried out using a custom script. These steps were wrapped in a Snakemake 7.22.0 48 pipeline and an R script (Rstudio 2021.9.0.351) that are available in Github: https://github.com/vihoikka/Cas10_prober. Software packages used in the pipeline were Hmmer 3.3.2, CD-hit 4.8.1, CRISPRCasTyper 1.8.0, Muscle 5.1, FastTree 2.1.11, R4.1.1).<br>Plasmid maps were generated using freeware Benchling software (https://benchling.com/)

For manuscripts utilizing custom algorithms or software that are central to the research but not yet described in published literature, software must be made available to editors and reviewers. We strongly encourage code deposition in a community repository (e.g. GitHub). See the Nature Portfolio guidelines for submitting code & software for further information.

Data

Policy information about availability of data

All manuscripts must include a data availability statement. This statement should provide the following information, where applicable:

- Accession codes, unique identifiers, or web links for publicly available datasets
- A description of any restrictions on data availability
- For clinical datasets or third party data, please ensure that the statement adheres to our policy

The genome sequences used to make figure 1a were downloaded from NCBI (https://www.ncbi.nlm.nih.gov) on 14/03/2023.
Mass spectrometry data are available on FigShare at the following address: https://doi.org/10.6084/m9.figshare.c.6646859.v1
Experimentally determined 3D protein structure coordinates are available at https://www.rcsb.org

Research involving human participants, their data, or biological material

Policy information about studies with human participants or human data. See also policy information about sex, gender (identity/presentation), and sexual orientation and race, ethnicity and racism.

Reporting on sex and gender	not applicable
Reporting on race, ethnicity, or other socially relevant groupings	not applicable
Population characteristics	not applicable
Recruitment	not applicable
Ethics oversight	not applicable

Note that full information on the approval of the study protocol must also be provided in the manuscript.

Field-specific reporting

Please select the one below that is the best fit for your research. If you are not sure, read the appropriate sections before making your selection.

☒ Life sciences ☐ Behavioural & social sciences ☐ Ecological, evolutionary & environmental sciences

For a reference copy of the document with all sections, see nature.com/documents/nr-reporting-summary-flat.pdf

Life sciences study design

All studies must disclose on these points even when the disclosure is negative.

Sample size	technical triplicates and biological duplicates or triplicates (as stated) were obtained for all measurements. This represents the accepted norm for a biochemical study, allowing standard deviation and mean to be calculated.
Data exclusions	No data were excluded
Replication	Duplicate or triplicate experiments were carried out, as stated for each experiment
Randomization	Randomization was not applied to the small biochemical datasets studied, in keeping with established norms for this type of study.
Blinding	Blinding was not applied to the small biochemical datasets studied, in keeping with established norms for this type of study.

Reporting for specific materials, systems and methods

We require information from authors about some types of materials, experimental systems and methods used in many studies. Here, indicate whether each material, system or method listed is relevant to your study. If you are not sure if a list item applies to your research, read the appropriate section before selecting a response.

Materials & experimental systems

n/a	Involved in the study
☐	☒ Antibodies
☒	☐ Eukaryotic cell lines
☒	☐ Palaeontology and archaeology
☒	☐ Animals and other organisms
☒	☐ Clinical data
☒	☐ Dual use research of concern
☒	☐ Plants

Methods

n/a	Involved in the study
☒	☐ ChIP-seq
☒	☐ Flow cytometry
☒	☐ MRI-based neuroimaging

Antibodies

Antibodies used

V5 tag monoclonal antibody was purchased from Thermofisher (cat. no. R960-25; clone SV5-Pk1)
anti-mouse IgG was purchased from LI-COR Biosciences, catalog: 926-32212, Lot No. D10811-15

Validation

Western blot analysis of V5 tag was performed by loading 20 μg of whole cell extracts of untransfected HEK-293 and HEK-293 transiently overexpressing V5- His-LacZ using Novex® NuPAGE® 4-12% Bis-Tris gel (Product # NP0321BOX), Xcell SureLock Electrophoresis system (Product # EI0002), Novex sharp Pre-stained Protein Standard (LC5800). Proteins were transferred to a PVDF membrane and blocked with 5% skim milk for 1 hour at room temperature. V5- His-LacZ was detected at ~117 kDa using V5 Tag Mouse Monoclonal Antibody (Product # R960-25) at a 1:1000 dilution in 2.5% skim milk at 4°C overnight on a rocking platform. Goat Anti-Mouse IgG - HRP Secondary Antibody (Product # 62-6520) at 1:4000 dilution was used and chemiluminescent detection was performed using Pierce™ ECL Western Blotting Substrate (Product # 32106).
The anti-mouse IgG antibody was isolated by affinity chromatography using antigens coupled to agarose beads. Based on ELISA, this antibody reacts with the heavy and light chains of mouse IgG and with the light chains of mouse IgM and IgA. This antibody was tested by ELISA and/or solid-phase adsorbed to ensure minimal cross-reaction with bovine, chicken, goat, guinea pig, horse, human, rabbit, and sheep serum proteins, but the antibody may cross-react with immuno-globulins from other species. The conjugate has been specifically tested and qualified for Western blot and In-Cell Western Assay applications.
